# The effect of systematic lower-limb rehabilitation training in elderly patients undergoing lumbar fusion surgery: a retrospective study

**DOI:** 10.18632/oncotarget.22746

**Published:** 2017-11-28

**Authors:** Si-Kai Liu, Yan-Li Song, Wen-Yuan Ding, Da-Long Yang, Lei Ma, Si-Dong Yang

**Affiliations:** ^1^ Department of Spinal Surgery, The Third Hospital of Hebei Medical University, Shijiazhuang, 050051, Hebei Province, China; ^2^ Hebei Provincial Key Laboratory of Orthopaedic Biomechanics, Shijiazhuang, 050051, Hebei Province, China

**Keywords:** intervertebral disc degeneration, elderly patients, lower-limb rehabilitation gymnastics, orthopedic nursing, postoperative rehabilitation

## Abstract

**Objectives:**

The purpose of this study was to explore the effect of systematic lower-limb rehabilitation training in elderly patients undergoing lumbar fusion surgery due to serious degenerative intervertebral disc diseases.

**Results:**

At the 1st week after surgery, clinical rehabilitation effect in intervention group was better regarding lower-limb muscle strength, lower-limb DVT, VAS score, and ODI, as compared with control group (all *p* < 0.05). During the first two weeks after surgery, satisfaction rate in intervention group was higher than that in control group. However, there was no significant difference at last follow-up after surgery when comparing intervention group to control group.

**Materials and Methods:**

We retrospectively collected medical records of elderly patients (aged ≥ 60 yrs) undergoing lumbar fusion surgery between 01/2013 and 01/2015 in our department. Some of the identified patients randomly underwent postoperative systematic training of lower-limb rehabilitation gymnastics (intervention group, *n* = 240), the others not (control group, *n* = 300). During postoperative period, intervention group received lower-limb rehabilitation gymnastics treatment for 3 months, but control group did not. All patients were routinely asked to return hospital for a check in the 1st postoperative week, as well as the 2nd week, the 1st month, and the 3rd month. Clinical outcomes were evaluated by scoring lower-limb muscle strength, detecting lower-limb deep venous thrombosis (DVT), visual analogue scale (VAS) score, lumbar JOA score, Oswestry disability index (ODI) questionnaire, and performing satisfaction survey.

**Conclusions:**

In early postoperative stage, systematic lower-limb rehabilitation training can effectively speed up the recovery, beneficial to reducing lower-limb DVT and increasing patient satisfaction rate.

## INTRODUCTION

Nowadays, lumbar fusion surgery is popular in treating degenerative disc diseases, especially for lumbar spinal diseases [1–3]. Despite surgical advances, adults undergoing lumbar spine surgery have poorer physical and mental health outcomes compared to the general population [4–6]. More specifically, up to 40% report persistent pain, functional disability and poor quality of life and 20% to 24% undergo a reoperation [7–9]. Surgeons routinely recommend physical therapy to speed up rehabilitation after spine surgery. Postoperative rehabilitation has been reported to improve outcome after disc surgery [10]. However, some studies [1, 2, 11] reported that postoperative rehabilitation had no significant influence in pain and self-rated disability as compared to no treatment/self-management after lumbar surgery including lumbar disc herniation and spinal stenosis patients. Several randomized trials have found no significant difference between standard physical rehabilitation and either no treatment or an educational booklet [1, 2, 12].

As the population ages, the events of degenerative disc diseases in elderly patients seem to increase. Thus, knowledge of the results of treatment in this age group is important to the orthopaedic surgeons. As we know, lumbar spinal fusion surgery is an effective method of treatment for such patients. However, elderly patients suffering from degenerative disc diseases are different from those younger patients with the same diseases. Elderly patients may experience longer on-bed time in a hospital after being performed a fusion operation, as compared to younger patients. In addition, it is another long term for the elderly patients to recover from an operation trauma, due to the aged body and weak physical function. Therefore, during that period, postoperative complications, such as deep venous thrombosis (DVT) and lower-limb muscle atrophy, are more likely to occur and increase. However, few studies have reported whether systematic lower-limb rehabilitation training in elderly patients is effective on this problem existing in the process of body recovery after lumbar fusion surgery.

Clinically, we have often asked the elderly patients undergoing lumbar fusion surgery to perform lower-limb rehabilitation gymnastics as a prophylaxis of postoperative complications. Thus, the aim of this study is to explore whether this method (lower-limb rehabilitation gymnastics) is effective on postoperative rehabilitation in elderly patients after lumbar fusion surgery.

## RESULTS

### Lower-limb muscle strength

As shown in Table 1, it was of no difference regarding preoperative comparison of lower-limb muscle strength (*χ* ^2^ = 0.443, *p* = 0.506). After spinal surgery, lower-limb muscle strength recovered much better as compared to the preoperative status (*p* < 0.001). However, there was no significant difference at last follow-up after surgery when comparing intervention group with control group regarding lower-limb muscle strength (*χ* ^2^ = 0.254, *p* = 0.614).

**Table 1:** Comparison of lower-limb muscle strength between intervention group and control group

Group	Preoperation		Po-1st wk			Po-2nd wk			Po-1st month			Po-3rd month		
Grade	III	IV	III	IV	V	III	IV	V	III	IV	V	III	IV	V
Control	54	246	26	195	79	18	198	84	1	121	178	0	18	282
(*n* ***=*** 300)														
Intervention	38	202	20	152	68	15	168	57	1	85	154	0	12	228
(*n* ***=*** 240)														

*P* < 0.001 by chi-square test, comparing postoperative lower-limb muscle strength to the preoperative status. Po-, postoperation.

### Lower-limb DVT

As shown in Table 2, during the first week after surgery, lower-limb DVT in intervention group was less than that in control group (*χ* ^2^ = 10.012, *p* = 0.002). However, there was no significant difference at last follow-up after surgery when comparing intervention group with control group regarding lower-limb DVT (*χ* ^2^ = 1.256, *p* = 0.262).

**Table 2:** Comparison of postoperative DVT

Group	Po-1st wk		Po-3rd month	
	DVT	Non-DVT	DVT	Non-DVT
Control	63	237	16	284
(*n* ***=*** 300)				
Intervention^* #^	26	214	8	232
(*n* ***=*** 240)				

^*^χ^2^ = 10.012, *p* = 0.002, by Pearson Chi-Square Test, compared with Control group, at the 1st week after surgery.

^#^*χ*^2^ = 1.256, *p* = 0.262, by Pearson Chi-Square Test, compared with Control group, at the 3rd month after surgery.

DVT, deep venous thrombosis; Po-, postoperation.

### VAS score

As shown in Table 3, at the first week after surgery, VAS score in intervention group was less than that in control group (*p* = 0.001, Student's *t* test). However, there was no significant difference on the time points of the 2nd week, the 1st month and the 3rd month after surgery when comparing intervention group with control group regarding VAS score (all *p* > 0.05).

**Table 3:** Comparison of VAS score by Student's t test

Group	Preoperative	1st wk	2nd wk	1st month	3rd month
Control	6.0 ± 2.1	3.8 ± 2.2	2.4 ± 1.1	1.8 ± 1.4	1.0 ± 0.2
Intervention	6.1 ± 2.5	3.2 ± 2.0	2.3 ± 1.2	1.9 ± 1.5	1.0 ± 0.3
*P*-value	0.614	0.001	0.314	0.425	> 0.99

VAS, Visual Analogue Scale.

### JOA score

As shown in Table 4, there was no significant difference on any time points after surgery when comparing intervention group with control group regarding VAS score (all *p* > 0.05).

**Table 4:** Comparison of JOA score by Student's t test

Group	Preoperative	1st wk	2nd wk	1st month	3rd month
Control	8.0 ± 1.2	14.2 ± 2.1	16.0 ± 2.7	19.0 ± 7.0	22.5 ± 6.5
Intervention	8.2 ± 1.5	3.2 ± 2.0	15.8 ± 2.5	20.0 ± 8.0	23.0 ± 6.0
*P*-value	0.086	0.080	0.377	0.122	0.359

OA, Japanese Orthopaedic Association.

### ODI score

As shown in Table 5, at the first week after surgery, ODI score in intervention group was less than that in control group (*p* = 0.016, Student's t test). However, there was no significant difference on the time points of the 2nd week, the 1st month and the 3rd month after surgery when comparing intervention group with control group regarding ODI score (all *p* > 0.05).

**Table 5:** Comparison of ODI by Student's t test

Group	Preoperative	1st wk	2nd wk	1st month	3rd month
Control	46 ± 22	32 ± 20	28 ± 14	16 ± 10	10 ± 4
Intervention	44 ± 21	28 ± 18	26 ± 16	18 ± 16	10 ± 8
*P*-value	0.285	0.016	0.122	0.076	> 0.99

ODI, Oswestry disability index.

### Satisfaction survey

As shown in Table 6, during the first two weeks after surgery, satisfaction rate in intervention group was higher than that in control group (*χ* ^2^ = 25.257, *p* < 0.001; *χ* ^2^ = 10.449, *p* = 0.005, respectively). However, there was no significant difference on the time points of the 1st month and the 3rd month after surgery when comparing intervention group with control group regarding satisfaction rate (*χ* ^2^ = 1.962, *p* = 0.375; *χ* ^2^ = 0.727, *p* = 0.695, respectively).

**Table 6:** Comparison of intervention group with control group regarding satisfaction survey

After surgery	Intervention group	Control group	Chi-square tests	
	Very satisfied/satisfied/dissatisfied	Very satisfied/satisfied/dissatisfied	*χ*^2^	*p*-value
1st wk	102 cases/114 cases/24 cases	95 cases/124 cases/81 cases	25.257	< 0.001
2nd wk	126 cases/105 cases/9 cases	160 cases/108 cases/32 cases	10.449	0.005
1st month	144 cases/90 cases/6 cases	162 cases/129 cases/9 cases	1.962	0.375
3rd month	148 cases/89 cases/3 cases	180 cases/118 cases/2 cases	0.727	0.695

## DISCUSSION

Currently, it is controversial on the effect of postoperative rehabilitation after surgery. A prospective study [1] of postoperative rehabilitation with lumbar spinal stenosis patients only found that routinely performed active physiotherapy with strengthening and stretching home exercises did not improve functional outcome (ODI) as compared with standard treatment. Neither rehabilitation program nor education booklet for the postoperative management had a significant impact on long-term outcome [12]. Recently, a randomized controlled trial [11] with 12-month follow-up indicated that quality of life and disability cannot be improved by active postoperative rehabilitation after spinal fusion surgery in patients with spondylolisthesis. However, the results from some other studies are to the contrary. Ostelo et al. [10] found that exercise programs starting 4 to 6 weeks postsurgery seemed to lead to a faster decrease in pain and disability than no treatment. High intensity exercise programs seemed to lead to a faster decrease in pain and disability than low intensity programs. But there were no significant differences between supervised and home exercises for pain relief, disability, or global perceived effect. Canbulat et al. [13] reported that VAS and ODI were both improved by a rehabilitation protocol for patients with lumbar degenerative disc diseases treated with lumbar total disk replacement. A randomized clinical trial [14] also found that the integrated programme of prehabilitation and early rehabilitation improved the outcome and shortened the hospital stay without more complications, pain or dissatisfaction.

Except for the postoperative rehabilitation training, some other factors may directly or indirectly influence the effect of postoperative rehabilitation after fusion surgery. One recent study [15] showed that the psoas muscle can be beneficial in overall postoperative rehabilitation with early ambulation and greater improvement in functional outcomes (VAS). Some findings [16, 17] supported that incorporating cognitive-behavioral strategies into postoperative physical therapy may address psychosocial risk factors and improve pain, disability, general health, and physical performance outcomes after surgery. Health behavior change counseling intervention also increased patient participation in physical therapy and/or home exercise programs, reduced disability, and improved health status after surgery for degenerative lumbar spinal stenosis [18].

It is true that elderly patients suffering from lumbar disc diseases are very different from those younger patients with the same diseases. Elderly patients are believed to experience longer hospital time after undergoing a fusion operation compared to younger patients. In addition, it is another long term for the elderly patients to recover from an operation trauma, due to the aged body and their own weak physical function. Therefore, during that period, postoperative complications, such as DVT and lower-limb muscle atrophy, are more likely to occur and increase. However, few studies have reported whether systematic lower-limb rehabilitation training in elderly patients is effective on this problem existing in the process of body recovery after lumbar fusion surgery. In our study, systematic lower-limb rehabilitation training (gymnastics) showed good effect on the rehabilitation after lumbar fusion surgery in the elderly patients, especially in the early stage postoperatively.

In clinical situations, we often asked and guided the patients to do exercise after an operation according to the procedures of lower-limb rehabilitation gymnastics. But we did not assess the effect then. Herein, this study has been designed and performed to evaluate the effect of lower-limb rehabilitation gymnastics on the functional recovery and complications developed during the process of functional recovery. As a consequence, in the early stage after surgery (during the first one or two weeks), lower-limb muscle strength, and patient satisfaction rate are better in intervention group when compared to control group. Besides, lower-limb DVT and ODI score are less in intervention group as compared to control group. However, there is no significant difference between the two groups regarding the above items evaluated in the later stage (on the timepoint of the 1st and the 3rd month). Regarding the VAS score reported in the current study, it has been found that lower-limb rehabilitation training/gymnastics does not increase back/leg pain, but relieves postoperative pain to a certain degree.

Surely, this study has indicated very important clinical significance. However, this work also has some limitations. Firstly, as a retrospective single-center case-control study, it lacks extensive representativeness. Secondly, we have not applied blind methods throughout the study. Thirdly, the sample size of patients included in the study is not large enough. So future research should strive to overcome these shortcomings, provide more reliable clinical research data. It is best to be a large sample, prospective, multicenter, randomized, controlled study, with blind methods applied.

In conclusion, in early postoperative stage, systematic lower-limb rehabilitation training can effectively speed up the recovery, beneficial to reducing lower-limb DVT and increasing patient satisfaction rate.

## MATERIALS AND METHODS

### Ethics statement

This study has been approved by Ethics Committee of the Third Hospital of Hebei Medical University. The approval number is K2017-05-02.

### Patients and inclusion criteria

**Figure 1:**
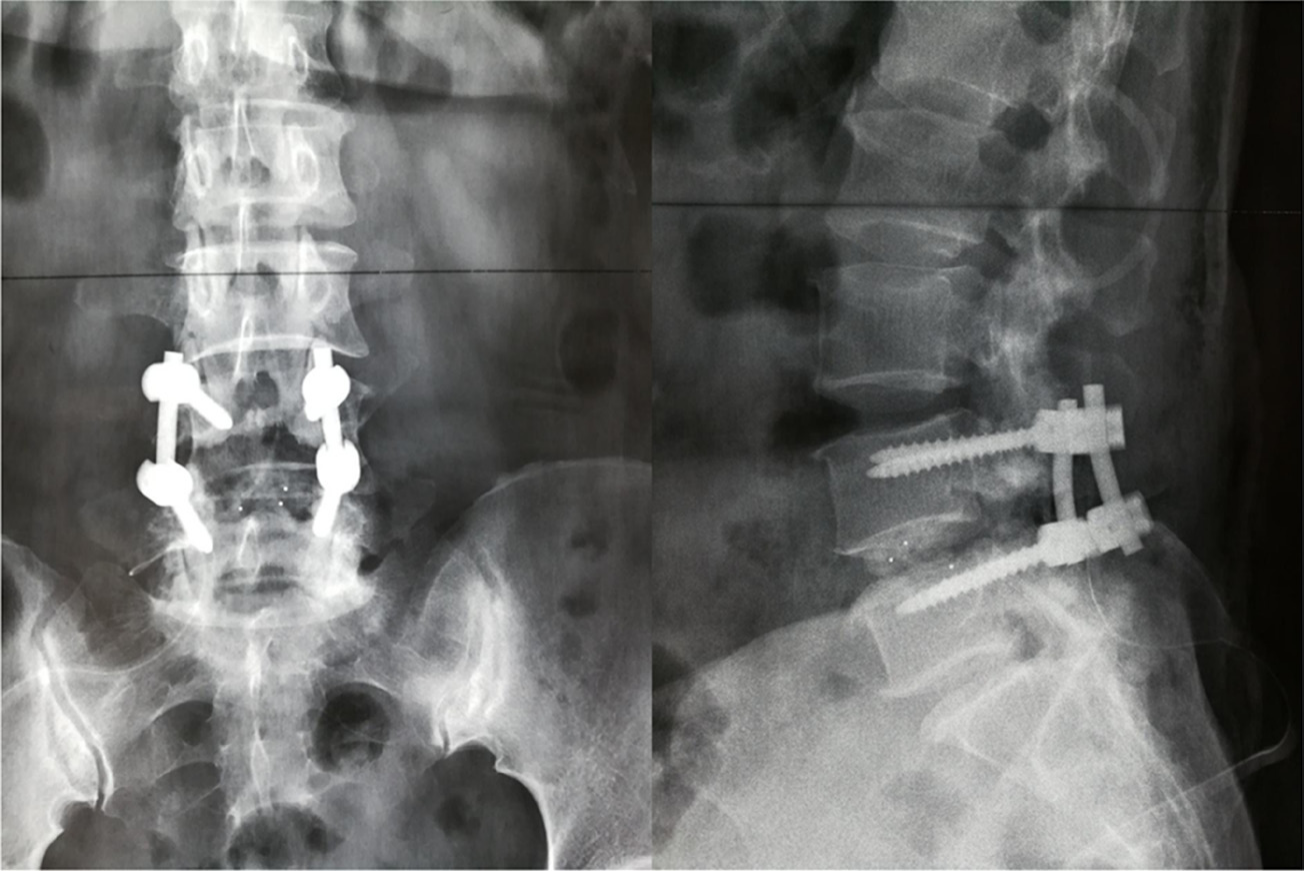
Postoperative X-ray image of lumbar fusion surgery

**Figure 2:**
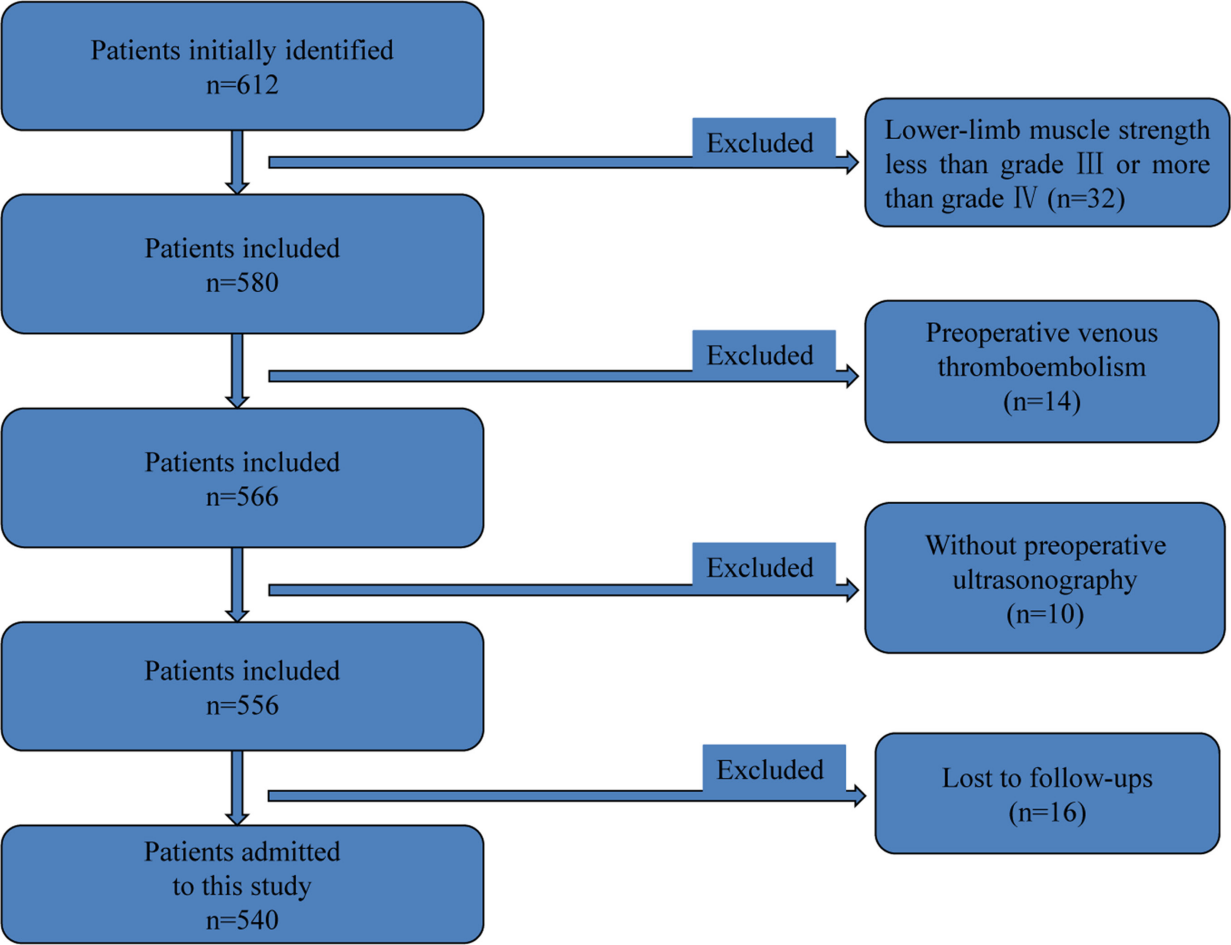
Flow diagram used for patient selection

We retrospectively collected medical records of elderly patients (aged ≥ 60 yrs) undergoing lumbar fusion surgery between 01/2013 and 01/2015 in our spinal department. Figure 1 shows the postoperative X-ray image of lumbar fusion surgery. Some of the identified patients randomly underwent systematic training of lower-limb rehabilitation gymnastics (intervention group), the others not (control group). As Figure 2 showed, 612 cases were initially identified, and finally 540 patients were included and admitted to this study. Inclusion criteria of the present study were as follows. 1- All patients have undergone lumbar spinal fusion surgery. 2- Preoperative lower-limb muscle strength was grade III or grade IV (because our lower-limb gymnastics is not suitable for the patients under grade II and meaningless to those grade V). 3- No history of other operations were on the lower-limbs. 4-No neuromuscular disease were on the limbs. 5-No pathologicalmyelitis or neuropathy existed. 6-All the patients have been excluded from lower-limb DVT by preoperative ultrasonography. Patients who did not have regular follow-up visits or had systemic disorders were also excluded.

### Intervention methods

The patients according to the treatment during that period were randomly divided into intervention group (*n* = 240) or control group (*n* = 300). In intervention group, there were 116 males and 124 females. The median age was 65 (IQR = 6) years old. In control group, there were 131 males and 169 females. The median age was 64 (IQR = 7) years old. During perioperative period, both intervention group and control group received same routine nursing care and drug treatment including low molecular weight heparin (LMWH) after surgery. During postoperative period, intervention group received lower-limb rehabilitation gymnastics treatment for 3 months, but control group did not. The lower-limb rehabilitation gymnastics was performed as follows. In the first section, let the patients lie flat with relaxation. In the second section, let them centripetally massage double lower limbs for 5 minutes. In the third section, ankle pump movement, let double foot try to flex or extend for 5 seconds, repeating 50 times per section, 3 sections each day. In the fourth section, knee-pressing motion, keeping legs straight, try to press knees down for 10 seconds, repeating 20 times per section, 3 sections each day. In the fifth section, let quadriceps stay static contraction and double lower limbs unbend; then try to let foot stand and lower limbs press down on bed, maintaining for 10 seconds, repeating the movement 20 times per section, 3 sections each day. In the last section, bend the knees and hip, and make the double knee joints flex for 30 degrees, keeping relaxed, repeating 20 times per section, 3 sections each day.

### Evaluation of rehabilitation effect

All patients were routinely asked to return hospital for a check in the 1st postoperative week, as well as the 2nd week, the 1st month, and the 3rd month, after surgery. Clinical rehabilitation effect was evaluated by checking lower-limb muscle strength, detecting the lower-limb DVT, visual analogue scale (VAS) score, Japanese Orthopaedic Association (JOA) score, Oswestry disability index (ODI) questionnaire, and performing satisfaction survey. Lower-limb muscle strength was graded according to the British medical research council's classification criteria. In addition, satisfaction survey was classified into three grades, very satisfied, satisfied and dissatisfied.

### Statistical analyses

Statistical analyses were performed using SPSS for Windows, version 18.0 (SPSS Inc., USA). All measurement data are presented as the mean±SD (standard deviation) when data satisfied criteria for normality with *p* > 0.10. Otherwise, it should be presented as median (interquartile range, IQR). When data satisfied criteria for normality and homogeneity of variance, statistical analysis between groups was performed using Student's t test. For count data, chi-square test was used for data analysis. Values for *p* < 0.05 were regarded as significant with two-tailed tests.

## Abbreviations

DVT: deep venous thrombosis
LMWH: low molecular weight heparin
VAS: visual analogue scale
ODI: Oswestry disability index
JOA: Japanese Orthopaedic Association
SD: standard deviation
ANOVA: analysis of variance
IQR: interquartile range
